# Is E-health Progressing Faster Than E-health Researchers?

**DOI:** 10.2196/jmir.8.3.e24

**Published:** 2006-09-29

**Authors:** Henry W. W. Potts

**Affiliations:** ^1^Centre for Health Informatics and Multiprofessional EducationRoyal Free & University College Medical SchoolLondonUK

**Keywords:** Internet interventions, Online treatment, Information services, Safety management, Research policy

## Abstract

Formal Internet interventions exist in a broad context of diverse online health resources, which share elements in common like information, advice and peer support. However, most online health resources are not created by healthcare professionals. Internet interventions need to be designed to “compete” in that wider context. The democratization of production and distribution is central to the transformative effect of the Internet on society, yet potentially conflicts with healthcare’s need for an evidence base and safe practice. This is a core challenge for healthcare on the Internet.

## E-health Consumers Are Ahead of E-health Researchers

In a paper in this issue of the *Journal of Medical Internet Research*, Ritterband and colleagues describe the directions being taken by the International Society for Research on Internet Interventions (ISRII) [1], offering a clearly signposted way ahead for research in this field. What they propose are things that clearly need to be done. Their privileging of randomized controlled trials as the gold standard methodology makes sense if Internet interventions are to continue to garner support from government agencies and healthcare bodies. Nonetheless, I argue here that their path is a narrow one. We should look further afield, both because that is our role as researchers but even more because patients and members of the public are racing ahead of us.

While evidence-based medicine (EBM) is an essential paradigm, we should not let the methodological tools of EBM railroad our thinking: the desire to do systematic reviews and meta-analyses should not obscure issues of heterogeneity between interventions and between studies [2]. Equally, an artificial categorization of what constitutes an Internet intervention (or previous jargon like “interactive health communication applications”) should not blind us to systems involving similar processes.

The typical Internet interventions described by Ritterband et al use the Internet as a delivery system for computer-based treatments. However, such systems are only reaching a fraction of the audience using the Internet for health purposes. The ingredients of Internet interventions – information, advice, peer group support, one-to-one contact – are available through a huge variety of websites, online groups, blogs, wikis, BitTorrents and more. If we expand from health into related lifestyle issues like nutrition, fitness, sex and relationships and parenthood, the number of resources increases yet further. Tens of millions of people have joined online support groups in the US alone [3], while many more people are accessing health information. The sites and groups concerned may have been set up by individual patients, charities, activist groups, commercial bodies (either selling a product or relying on advertising), or healthcare professionals. The overwhelming majority of all these resources are informal, untested and without clinical input, quite unlike the Internet interventions supported by the ISRII.

## Competition

Faced with this diversity, health consumers are making choices about what resources to use. While clinicians value EBM quality criteria to support such choices, these are not used by the public [4]. Whether we like it or not, online health consumers are using untested, amateur resources or commercial sites with financial motivations. Moreover, they appear to be making greater use of such resources than 5 years ago [5]. Many of these amateur websites, online groups, blogs and all, seem valuable and safe, but is there enough ongoing research to demonstrate that?

The implication of this world of abundance of health-related information and peer support is that formal Internet interventions face competition, something not mentioned in the “directions” article [1]. The ISRII want to make Internet interventions available to “anyone, anywhere”: a laudable goal, but simply making them available will not be enough. The online consumer is not a passive recipient: they shop around, they try multiple sources, they review what they find and use others’ online reviews to guide their choices [6]. Online healthcare consumers are not assessing what they find against the EBM quality criteria on which formal Internet interventions score well, while some commercial sites have huge resources to spend on promotion and site design. This is the context that contributes to the low adherence that Ritterband and colleagues and others have described [1, 7]. We need Internet interventions that are not only good (effective and reliable), but which can compete for the online healthcare consumer’s attention and recognize that individuals will use multiple sites and in ways of their own choosing. Among other things, that means that Internet interventions should be free at the point of use, a tough economic model to achieve.

People want choice. Consider for example online support groups in cancer. There are online groups for very many types of cancer (far more than could ever exist as face-to-face groups), but the variety does not stop there. For more common cancers, people fragment in different ways, so there are groups for specific varieties, stages and treatments and groups for different sorts of people by locale, age, religion and more (*e.g.* war veterans with prostate cancer, lesbians with breast cancer, even belly dancers with breast cancer). There are multiple groups with apparently the same coverage. Internet interventions need to be equally adaptable and diverse. I predict that there will be demand for a huge variety of Internet interventions tailored in all sorts of ways, just as people generally want personalized health information [5].

## Democratization of Production

Away from healthcare, the Internet has been revolutionary and transformational because it has democratized production and distribution [6]. Traditional healthcare, given its safety critical context, utilises an evidence base and a process of risk management that generally involves some sort of governance. These are conflicting trends: the great value of the Internet is how easy it is to make material available, but the strictures of safety and proof of efficacy run counter to that. How do we garner the benefits of the Internet – the democratization of production and distribution that has produced so much content – while maintaining safe and good practice? The answer remains unclear, but it is a question the research community should address. Traditional Internet interventions, with content by healthcare professionals, prescribed to patients, can only be part of the answer. We should recognize the value and popularity of user-generated content in non-health contexts [6] and work out how to better integrate it into online healthcare resources.

The challenges of the online environment for healthcare have been considered before [8]. There are uncounted online resources, amateur as well as professional, that overlap with formal Internet interventions. ISRII might have to address the implications of this context more explicitly, as others have done [9, 10]. Beyond healthcare, there are many more innovations that draw on user-generated content and the Internet’s democratization of production and distribution. The “killer application” in e-health will perhaps be something that can marry the democratized nature of MySpace or Wikipedia with the safety critical nature of healthcare.

## Abbreviations

EBM: Evidence-Based Medicine
